# Epigenetic dysregulation-induced metabolic reprogramming fuels tumor progression in bladder cancer

**DOI:** 10.3389/fmolb.2025.1602700

**Published:** 2025-06-23

**Authors:** Jian Zhang, Xiaosong Fan, Xu Xu, Yichao Han, Weixing Yu, Bicheng Yang, Yanling Chen, Shaolin Zhang

**Affiliations:** ^1^ Department of Urology, Shangyu People’s Hospital of Shaoxing, Shaoxing University, Shaoxing, Zhejiang, China; ^2^ Hangzhou Center for Health Development, Hangzhou, Zhejiang, China; ^3^ Department of Urology, The First Affiliated Hospital, Sun Yat-sen University, Guangzhou, Guangdong, China; ^4^ Digestive Endoscopy Center, The First Affiliated Hospital of Wannan Medical College (Yijishan Hospital of Wannan Medical College), Wuhu, Anhui, China; ^5^ Department of Neurosurgery, The First Affiliated Hospital of Wannan Medical College (Yijishan Hospital of Wannan Medical College), Wuhu, Anhui, China

**Keywords:** bladder cancer, metabolic reprogramming, tumor aggressiveness, inflammation, DNA methylation, USF2, NuRD complex

## Abstract

**Background:**

Bladder cancer remains a significant global health challenge with a high mortality rate despite advancements in treatment modalities. Metabolic alterations serve as crucial contributors to cancer progression, particularly influencing tumor aggressiveness and patient outcomes. Therefore, this study aimed to identify and characterize metabolic hubs associated with disease progression and tumor aggressiveness in bladder cancer.

**Methods:**

DNA methylation, mRNA expression and protein expression, along with clinical data for bladder cancer patients were retrieved from TCGA database. Differentially expressed metabolic hubs among tumor aggressiveness groups and between early vs advanced stage tumors were identified using ANOVA and Student’s *t*-test respectively, whereas survival association of metabolic genes was assessed using an R code. Pathway enrichment, network construction, random walk, transcription factor prediction and gene set enrichment analyses were conducted using DAVID, Cytoscape, Java, ChEA3 and GSEA tools respectively. Validation of the identified gene signature was performed using NCBI GEO datasets.

**Results:**

Through a metabolism-targeted differential expression and survival analysis-based approach, we identified 105 metabolic genes, whose expression patterns correlated with tumor aggressiveness and clinical outcomes in bladder cancer patients. Subsequent network construction and random walk analysis refined this list to a seven-gene metabolic signature (Metab-GS), comprising both oncogenic (ALDH1B1, ALDH1L2, CHSY1, CSGALNACT2, GPX8) and tumor suppressors (FBP1, HPGD) hubs. Upstream analysis identified epigenetic modifications, particularly DNA hypermethylation of tumor suppressor metabolic hubs and reduced USF2-NuRD complex activity-driven increased expression of oncogenic metabolic hubs, contributing to glycolytic shift and extracellular matrix remodeling, and establishing an inflammatory tumor microenvironment. Lastly, validation of our findings in multiple independent GEO datasets confirmed that high Metab-GS scores are associated with tumor aggressiveness and progression, advanced disease stage, metastatic spread, disease recurrence, and poor overall and cancer-specific survival in bladder cancer patients.

**Conclusion:**

Overall, a seven-gene metabolic signature predicts tumor aggressiveness and poor prognosis in bladder cancer patients, underscoring the potential of targeting the epigenetic dysregulation-induced metabolic reprogramming as a therapeutic strategy for aggressive bladder cancer.

## 1 Introduction

Cancer, encompassing a wide variety of tumor types, remains a formidable challenge that continuously propels advancements in biomedical research (Soerjomataram and Bray, 2021). Among these malignancies, bladder cancer, which originates in the urinary tract, is recognized as the 10th most frequently diagnosed cancer worldwide, with annual figures exceeding 600,000 new diagnoses and roughly 220,000 fatalities (Bray et al., 2024). The majority of patients are initially identified with non-muscle invasive bladder cancer, a stage often managed through localized therapeutic interventions or vigilant monitoring (Campistol and Lozano, 2025). For those with disease confined to the bladder without metastasis, standard treatment protocols typically involve surgical removal, either alone or combined with radiation and chemotherapy (Maisch et al., 2021). In contrast, when bladder cancer is deemed unresectable or has spread beyond the primary site, platinum-based chemotherapy regimens are the predominant systemic treatment option. Despite these approaches, the median survival duration for patients remains dishearteningly short, averaging around 14 months (Kwon and Lee, 2024; Moussa and Campbell, 2024). Recent developments in immunotherapy, specifically immune checkpoint inhibitors, have introduced new hope by improving survival in certain patient subsets, though overall response rates remain modest (Mancini et al., 2021). Addressing the substantial gaps in our molecular-level comprehension of bladder cancer is essential, as these knowledge deficits limit the advancement of more effective treatments and contribute to unfavorable patient outcomes. Hence, identifying actionable molecular pathways involved in tumor initiation, progression, aggressiveness, and clinical prognosis is a pressing priority (Soria et al., 2019; Flores Monar et al., 2023). Such insights could pave the way for more precise diagnostic tools and targeted therapeutic strategies, ultimately improving the prognosis and survival rates for bladder cancer patients.

Cancer is widely recognized to exhibit metabolic reprogramming as a core molecular hallmark (Hanahan, 2022). These alterations are not passive occurrences; they actively stimulate unchecked tumor cell proliferation and significantly affect disease progression, treatment outcomes, and patient survival (Martínez-Reyes and Chandel, 2021). Moreover, metabolic reprogramming also enable cancer cell survival in nutrient-deprived tumor microenvironments (Nong et al., 2023). Recent studies have delved into the complex metabolic reconfigurations that underpin bladder cancer development. Despite advancements, such as identifying disrupted glucose metabolism, onset of Warburg effect, and dysregulated lipid metabolism as critical biomarkers in bladder cancer (Burns et al., 2021; Zhu et al., 2021; Afonso and Gonçalves, 2023), certain gaps persist; such as the need to pinpoint key metabolic drivers linked to tumor aggressiveness and progression of these tumors; and the requirement for comprehensive metabolic biomarkers and signatures that can provide more accurate prognostic insights for bladder cancer patients (Pereira and Domingues, 2024). Moreover, deciphering the intricate interplay between metabolic alterations and the molecular mechanisms that drive tumor aggressiveness is critical. Gaining a deeper understanding of these processes is essential for developing targeted therapies that exploit the inherent vulnerabilities of bladder cancer.

Recent advancements in high-throughput expression profiling methods, coupled with access to vast datasets, have enabled researchers to rapidly uncover the molecular foundations of diseases. This progress has significantly enhanced our insight into disease pathogenesis and facilitated the discovery of promising targets for diagnosis, prognosis, and therapeutic strategies (Tirosh and Suvà, 2019; Sheng et al., 2020). In this study, we aimed to identify metabolic bases of tumor aggressiveness in bladder cancer and adopted a robust, metabolism-focused strategy that integrates tumor aggressiveness-related differential expression with survival analyses to dissect the metabolic processes underlying bladder cancer by leveraging publicly accessible transcriptomic data.

## 2 Materials and methods

### 2.1 Data retrieved

Gene expression, DNA methylation and protein expression data for bladder cancer patients were obtained from The Cancer Genome Atlas (TCGA) via the Broad GDAC Firehose portal (https://gdac.broadinstitute.org/). In addition, TCGA mutation data was retrieved from the cBioPortal platform (https://www.cbioportal.org/). In parallel, bladder cancer expression datasets were collected from the GEO database, specifically from the following studies: GSE13507 (Kim et al., 2010), GSE31684 (Riester et al., 2012), GSE32548 (Lindgren et al., 2012), GSE48075 (Guo et al., 2020), GSE83586 (Sjödahl et al., 2017), GSE120736 (Song et al., 2019), GSE124305 (Seiler et al., 2019), and GSE128959 (Sjödahl et al., 2020) to validate the findings.

### 2.2 Patients’ tumor aggressiveness classification

Epithelial-to-mesenchymal transition (EMT) is widely recognized as a key indicator of tumor aggressiveness and metastatic potential (Huang et al., 2022). In this study, we sourced the hallmark_EMT gene set from the Gene Set Enrichment Analysis (GSEA) portal (https://www.gsea-msigdb.org/). We then calculated Z-scores for each of the 200 genes in the signature across all patient samples. These individual Z-scores were aggregated to generate a comprehensive tumor aggressiveness score for every patient. Finally, patients were ranked by their scores and evenly divided into three groups: low, intermediate, and high aggressiveness.

### 2.3 Survival analyses

Kaplan-Meier survival plots for each gene in TCGA dataset were created using R. Patients lacking survival time or event data were omitted from the analysis. The optimal cut-off threshold was applied to stratify the groups, and survival differences between the groups were evaluated using the Log-rank (Mantel-Cox) test. A p-value of less than 0.05 was considered statistically significant.

### 2.4 Identification of differentially expressed metabolic targets

An extensive catalog of metabolic enzymes (Supplementary Table S1) was assembled by extracting gene lists from two primary resources: the Mammalian Metabolic Enzyme Database (https://hpcwebapps.cit.nih.gov/ESBL/Database/MetabolicEnzymes/MetabolicEnzymeDatabase.html) and the metabolic enzymes listed in the Kyoto Encyclopedia of Genes and Genomes (KEGG) Pathway Database (https://www.genome.jp/kegg/pathway.html). The mRNA levels of these metabolic genes were compared across groups with varying tumor aggressiveness and stage using data from TCGA database. A False Discovery Rate (FDR) threshold of <0.05 was applied to determine significance. Additionally, survival analyses were conducted for each metabolic gene. Genes that met all of the following criteria: showing a consistent pattern of up- or downregulation across increasing tumor aggressiveness, being differentially expressed between early and advanced stage tumors, and having a significant association with survival outcomes, were designated as differentially expressed metabolic targets in bladder cancer.

### 2.5 Pathway enrichment analysis

Pathway enrichment analysis was conducted at DAVID functional annotation tool (https://david.ncifcrf.gov/summary.jsp). In brief, the list of differentially expressed metabolic targets was uploaded to determine their associated KEGG and Reactome pathways as well as their roles in Gene Ontology categories, including Biological Processes, Cellular Compartment and Molecular Functions, and a p-value of less than 0.05 was used to define statistical significance.

### 2.6 Network construction and random walk analysis

The list of identified differentially expressed metabolic targets was uploaded to the STRING database (https://cn.string-db.org/), from which an interaction file was downloaded and subsequently imported into Cytoscape 3.7.1 to construct the network. A custom Java code was then used to perform random walk analysis: Each node was initially allocated the same energy level, which was then redistributed across the network over several iterations. This process continued until the system reached equilibrium, at which point the amount of energy retained by each node was defined as its random walk (RW) score. To identify key metabolic hubs, we examined the distribution of RW scores and selected nodes with scores ≥2. This cutoff was chosen to yield a concise yet biologically meaningful signature that balances specificity and coverage of critical metabolic regulators in bladder cancer.

### 2.7 Gene set enrichment analysis (GSEA)

To quantify tumor aggressiveness, we calculated a score for each patient in the TCGA database by summing the Z-scores of gene expression levels from the hallmark_EMT gene set. Metab-GS scores were calculated by subtracting the sum of expression Z-scores of tumor suppressor differentially expressed metabolic targets from the sum of expression Z-scores of oncogenic differentially expressed metabolic targets for each patient in TCGA database. USF2-NuRD complex score was calculated by summing the expression Z-score of USF2 and genes involved in NuRD-complex for each patient in TCGA database. Patients were divided on the bases of (1) Metab-GS (low vs. high), (2) tumor aggressiveness scores (low vs. high), (3) USF2 expression (low vs. high), or (4) USF2-NuRD complex score (low vs. high). GSEA was performed using gene sets related to (1) bladder cancer, (2) NuRD complex-regulated gene expression, (3) Metabolic reprogramming, (4) tumor progression, and (5) cancer hallmarks downloaded from the GSEA website (http://software.broadinstitute.org/gsea/index.jsp).

### 2.8 Transcription factor prediction analyses

Transcription factor prediction for mRNAs was performed at freely available ChEA3 database (https://maayanlab.cloud/chea3/), where oncogenes from Metab-GS were uploaded and list of significantly predicted transcription factors was downloaded along with number of predicted targets from the uploaded list. Number of predicted USF2 binding sites in genes from Metab-GS were assessed from ChIPBase v.3 database (https://rnasysu.com/chipbase3/). USF2 binding scores for genes in Metab-GS were collected from Cistrome database (http://cistrome.org/db/#/). Briefly, as data was not available for any bladder cancer source at Cistrome database, USF2 binding score data for five different cancer cell lines was downloaded and averaged to get the binding scores for genes in Metab-GS.

### 2.9 Statistical analysis

For comparisons between two groups, we applied Student’s t-test, while one-way ANOVA followed by Tukey’s *post hoc* test was used to evaluate differences across multiple groups. Pearson’s correlation coefficient was calculated to assess the relationships between variables. Bar graphs, dot plots, forest plots, and survival plots were generated using GraphPad Prism v6 (https://www.graphpad.com). Illustration was created with BioRender (https://www.biorender.com), and Venn diagrams was constructed using an online tool (http://bioinformatics.psb.ugent.be/webtools/Venn).

## 3 Results

### 3.1 Identification of differentially expressed metabolic targets and pathways associated with tumor aggressiveness in bladder cancer

**FIGURE 1:**
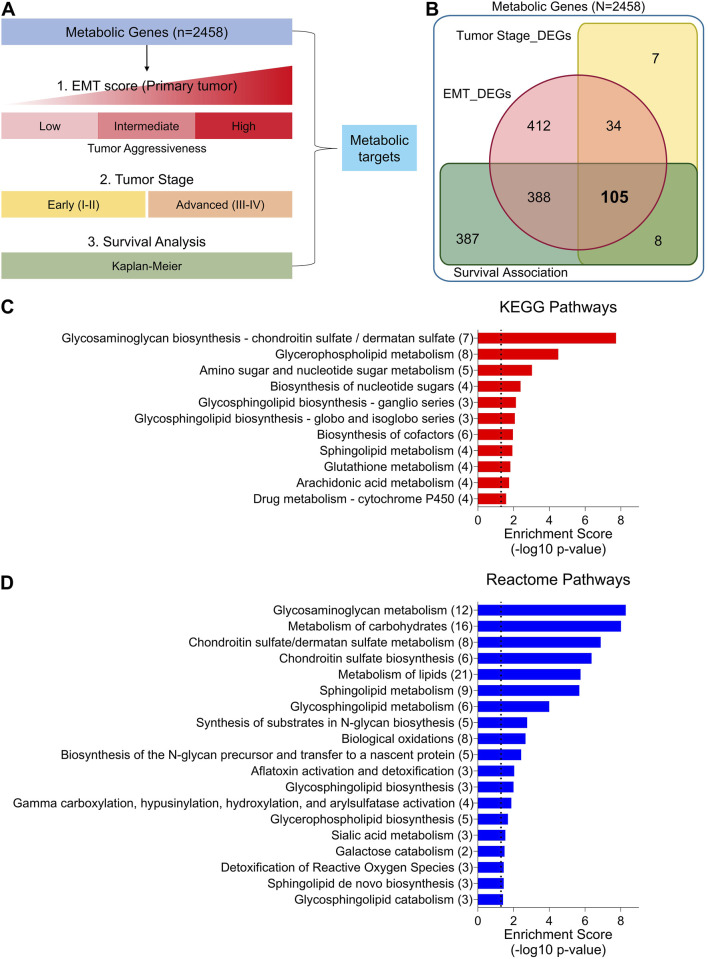
Identification of differentially expressed metabolic targets and pathways associated with tumor aggressiveness in bladder cancer. **(A)** Illustration showing analysis pipeline used to identify differentially expressed metabolic targets in bladder cancer from TCGA database. Briefly, metabolic gene expression was compared (1) among EMT score based tumor aggressiveness groups (low, intermediate, high), and (2) between early and advanced stage tumors, as well as (3) their association with patient survival was checked. **(B)** Venn diagram showing number of metabolic targets identified through pipeline in **(A)**. **(C)** Bar-graph showing results of KEGG pathway enrichment analysis using common metabolic targets identified in **(A,B)**. **(D)** Bar-graph showing results of Reactome pathway enrichment analysis using common metabolic targets identified in **(A,B)**. EMT: epithelial-to-mesenchymal transition, DEGs: Differentially expressed genes.

In order to identify differentially expressed metabolic targets associated with disease progression, tumor aggressiveness and survival, we first compiled a catalog of all the genes annotations associated with metabolic enzymes (For details: see Materials and Methods section) and downloaded the gene expression data of bladder cancer patients from TCGA database. We then carried out differential expression analysis of metabolic genes under two conditions: (1) across tumor groups categorized by low, intermediate, and high aggressiveness based on EMT scores (details provided in the Materials and Methods section), and (2) between early-stage and advanced-stage tumors. Additionally, we evaluated the association of each metabolic gene with patient survival using Kaplan-Meier analysis across TCGA bladder cancer data (Figure 1A). Through these analyses, we identified 105 genes that met the following criteria: (1) showed consistent up- or downregulation from less to more aggressive tumor groups, (2) were differentially expressed between early and advanced stages, and (3) exhibited a significant correlation with patient survival outcomes (Figure 1B).

Next, we performed pathway enrichment analyses identifying a range of metabolic processes associated with tumor progression and patient survival in bladder cancer, with glycosaminoglycan metabolism as top hit in both KEGG (Figure 1C) and reactome pathways (Figure 1D). Other enriched pathways can be attributed to nucleotide metabolism, carbohydrate metabolism, lipid metabolism, drug metabolism, and oxidative damage response (Figures 1C,D). GO Functional annotations on ‘biological processes’ and ‘molecular functions’ also identified terms related to above-mentioned metabolic processes, along with enrichment in Golgi membrane, endoplasmic reticulum and mitochondrial ‘cellular compartments’ (Supplementary Figures S1A–C). Overall, these findings highlight that diverse metabolic processes are rewired during the course of tumor progression in bladder cancer.

### 3.2 A metabolic gene signature determines aggressive disease state and poor survival in bladder cancer

**FIGURE 2:**
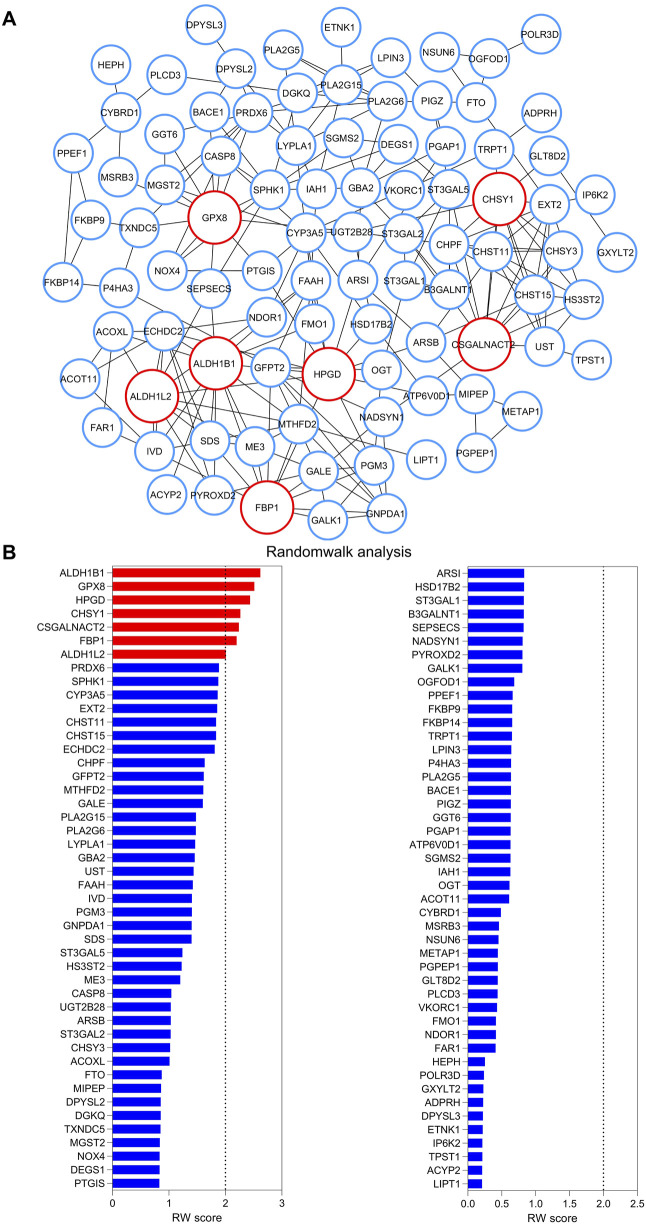
Network construction and random walk analysis. **(A)** Illustration showing interaction network of metabolic targets identified in Figures 1A,B. **(B)** Bar-graph showing results of random walk analysis of interaction network from **(A)**. Genes are sorted from high to low random walk (RW) score and top seven metabolic hubs identified through random walk analysis are highlighted in Red in **(A)** as well.

**FIGURE 3:**
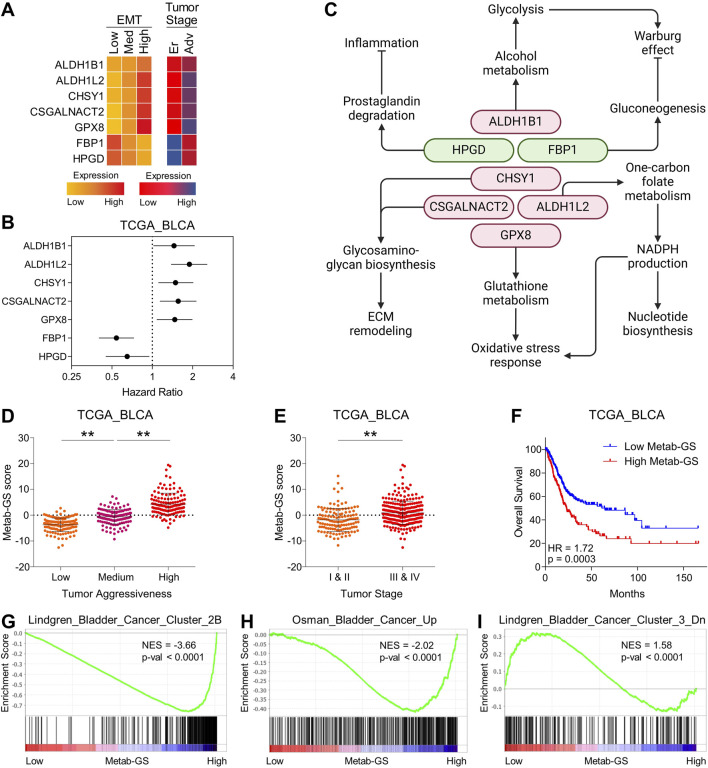
A metabolic gene signature determines aggressive disease state and poor survival in bladder cancer. **(A)** Heatmap showing changes in expression of top seven metabolic hubs among EMT-based low to high tumor aggressiveness groups (left) and between early and advanced tumor stage (Right) in bladder cancer from TCGA database. **(B)** Forest-plot showing association of top seven metabolic hubs with patient survival in bladder cancer from TCGA database. **(C)** Illustration showing molecular mechanisms regulated by identified seven metabolic hubs associated with tumor aggressiveness and patients’ survival in bladder cancer. Tumor suppressor metabolic hubs are shown in Green whereas oncogenic ones are shown in Red. **(D)** Dot-plot showing changes in Metab-GS score among low, medium and highly aggressive tumor groups in bladder cancer patients from TCGA database. **(E)** Dot-plot showing changes in Metab-GS score between early and advanced stage tumors in bladder cancer patients from TCGA database. **(F)** Kaplan-Meier survival plot showing overall survival analysis based on low and high Metab-GS score in bladder cancer patients from TCGA database. **(G–I)** GSEA showing the enrichment of bladder cancer-related gene sets, Lindgren_Bladder_Cancer_Cluster_2B **(G)**, Osman_Bladder_Cancer_Up **(H)**, and Lindgren_Bladder_Cancer_Cluster_3_Dn **(I)** between low and high Metab-GS score expressing tumors in bladder cancer patients from TCGA database. Up, upregulated; Dn, downregulated; NES, normalized enrichment score. **p < 0.01.

To pinpoint critical metabolic hub genes, we initially built an interaction network using the STRING database and observed that 92 out of the 105 candidate genes formed a connected network (Figure 2A). Next, we applied a random walk analysis on this network to prioritize key metabolic genes. Genes with an aggregated random walk score of ≥2, indicating higher energy retention at steady state, were considered top candidates (Figure 2B) (For details: see Materials and Methods section), suggesting that enzymes in this metabolic gene signature (hereon, termed as Metab-GS) serves as key metabolic hubs driving tumor progression in bladder cancer. Our Metab-GS comprises of seven enzymes, namely, aldehyde dehydrogenase 1 family member B1 (ALDH1B1), aldehyde dehydrogenase 1 family member L2 (ALDH1L2), chondroitin sulfate synthase 1 (CHSY1), chondroitin sulfate N-acetylgalactosaminyltransferase 2 (CSGALNACT2), fructose-bisphosphatase 1 (FBP1), glutathione peroxidase 8 (GPX8), and 15-hydroxyprostaglandin dehydrogenase (HPGD) (Figure 2). Notably, five of these metabolic hubs (ALDH1B1, ALDH1L2, CHSY1, CSGALNACT2, GPX8) (1) demonstrated a consistent increase in expression from low to highly aggressive tumor groups (Figure 3A; Supplementary Figures S2A–E), (2) were significantly upregulated in advanced-stage tumors relative to early-stage ones (Figure 3A; Supplementary Figures S2H–L), and (3) and showed a strong association with poorer survival outcomes in bladder cancer patients (Figure 3B; Supplementary Figures S2O–S), highlighting their potential roles as oncogenic metabolic drivers. In contrast, two of these metabolic hubs (FBP1 and HPGD) (1) were progressively downregulated across increasing tumor aggressiveness (Figure 3A; Supplementary Figures S2F,G), (2) exhibited lower expression in advanced-stage tumors compared to early-stage ones (Figure 3A, and Supplementary Figures S2M,N), and (3) and were linked to more favorable survival outcomes (Figure 3B; Supplementary Figures S2T,U), suggesting tumor-suppressive metabolic functions in bladder cancer.

Notably, the perturbations in Metab-GS leads to diverse cancer-promoting mechanisms. For instance, upregulation of ALDH1B1 promotes the glycolysis-dependent Warburg effect (Tsochantaridis et al., 2024), while inhibition of FBP1-driven gluconeogenesis further shifts cellular metabolism towards glycolysis (Xu et al., 2022). Upregulated ALDH1L2 facilitates one-carbon folate metabolism to produce NADPH, which not only acts as a reducing agent to promote nucleotide biosynthesis, but also enhances antioxidant defenses (Lee and Vousden, 2024), along with elevated GPX8-driven glutathione metabolism that helps manage oxidative stress responses crucial for tumor cell survival (Li et al., 2022). Increased CHSY1 and CSGALNACT2 expression remodels the extracellular matrix (ECM) through glycosaminoglycan biosynthesis (Zeng et al., 2018; Wu et al., 2021), whereas downregulation of HPGD induces prostaglandin-driven inflammation (Sun et al., 2021), both of which are critical for tumor progression. In line with this, our Metab-GS was associated with tumor aggressiveness (Figure 3D), advanced tumor stage (Figure 3E) and poor survival (Figure 3F) in bladder cancer patients. Furthermore, genes that are upregulated in bladder cancer were found to be significantly enriched in tumors with high Metab-GS scores compared to those with low scores (Figures 1G,H). Conversely, genes downregulated in bladder cancer were predominantly enriched in tumors with low Metab-GS scores relative to those with high scores (Figure 1I). These findings support the conclusion that the metabolic alterations identified are closely linked to tumor aggressiveness in bladder cancer.

### 3.3 DNA hypermethylation is associated with reduced expression of tumor suppressor metabolic hubs during disease progression in bladder cancer

**FIGURE 4:**
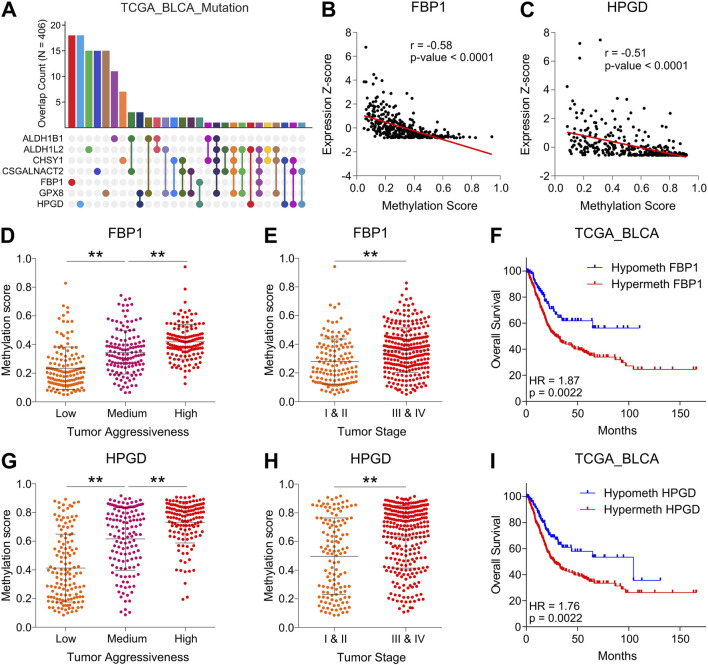
DNA hypermethylation is associated with reduced expression of tumor suppressor metabolic hubs during disease progression in bladder cancer. **(A)** Bar-graph showing number of patients having individual or co-mutations of seven metabolic hubs in bladder cancer patients from TCGA database. **(B,C)** Dot-plot showing correlation between expression Z-score and DNA methylation score of FBP1 **(B)** and HPGD **(C)** in bladder cancer patients from TCGA database. **(D)** Dot-plot showing changes in methylation score of FBP1 among low, medium and highly aggressive tumor groups in bladder cancer patients from TCGA database. **(E)** Dot-plot showing changes in methylation score of FBP1 between early and advanced stage tumors in bladder cancer patients from TCGA database. **(F)** Kaplan-Meier survival plot showing survival analysis based on low and high methylation score of FBP1 in bladder cancer patients from TCGA database. **(G)** Dot-plot showing changes in methylation score of HPGD among low, medium and highly aggressive tumor groups in bladder cancer patients from TCGA database. **(H)** Dot-plot showing changes in methylation score of HPGD between early and advanced stage tumors in bladder cancer patients from TCGA database. **(I)** Kaplan-Meier survival plot showing survival analysis based on low and high methylation score of HPGD in bladder cancer patients from TCGA database. **p < 0.01.

Next, we aimed to find that how this Metab-GS is regulated during the course of tumor progression in bladder cancer. To this end, we first checked the mutation profiles of metabolic genes from Metab-GS and found that all these genes were not mutated much in bladder cancer tissues (Figure 4A). Epigenetic dysregulation, which alters gene expression without changing the DNA sequence, has been strongly implicated in cancer progression. DNA hypermethylation typically results in gene silencing, whereas DNA hypomethylation is often associated with enhanced gene transcription (Yu et al., 2024). To this end, we investigated the correlation between DNA methylation and expression of genes from Metab-GS in bladder cancer, and found high inverse correlation (>−0.50) between DNA methylation and expression of tumor suppressor metabolic hubs, FBP1 and HPGD (Figures 4B,C). On the other hand, the correlations between DNA methylation and expression of oncogenic metabolic hubs from Metab-GS, were not very high (Supplementary Figures S3A–E). In addition, FBP1 and HPGD gene loci (1) were successively hypermethylated from low to high aggressive tumor groups (Figures 4D,G), and (2) were hypermethylated in advanced stage tumors compared to early stage ones (Figures 4E,H). Notably, the hypermethylation at these loci was also associated with poor survival in bladder cancer patients (Figures 4F,I). On the other hand, the oncogenic metabolic hub gene loci were not either (1) successively hypomethylated from low to high aggressive tumor groups (Supplementary Figures S3F–J), (2) hypomethylated in advanced stage tumors compared to early stage ones (Supplementary Figures S3K–O) nor (3) their hypomethylation was associated with poor survival for all of the oncogenic metabolic hubs (Supplementary Figures S3P–T). Overall, these results suggest that DNA hypermethylation at FBP1 and HPGD loci is associated with their reduced expression, aligning with disease aggressiveness and poor survival in bladder cancer patients.

### 3.4 Suppressed USF2-NuRD complex-driven histone deacetylation is associated with increased expression of oncogenic metabolic hubs during disease progression in bladder cancer

**FIGURE 5:**
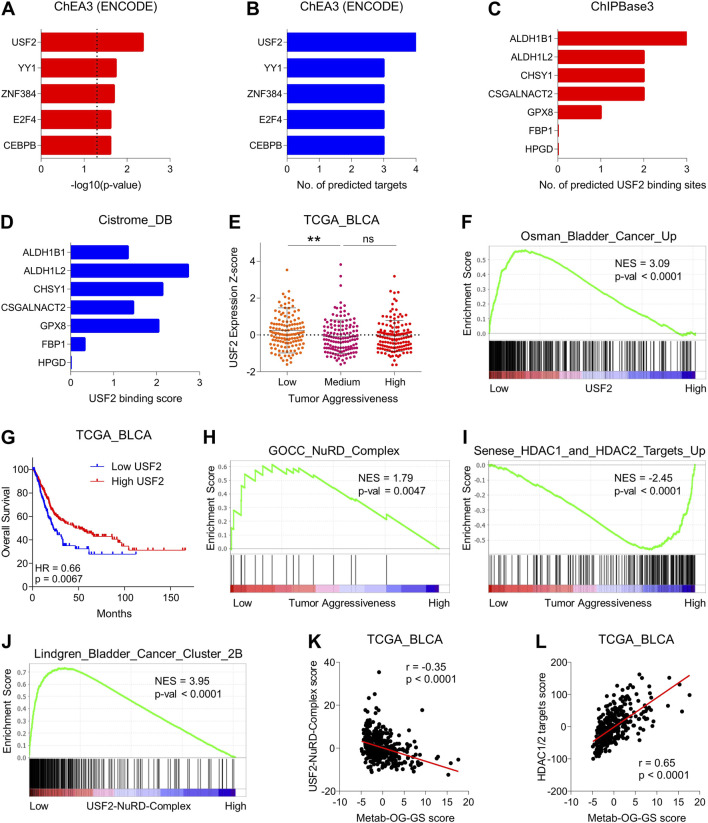
Suppressed USF2-NuRD complex-driven histone deacetylation is associated with increased expression of oncogenic metabolic hubs during disease progression in bladder cancer. **(A)** Bar-graph showing significance of transcription factors to regulate oncogenic metabolic hubs in bladder cancer predicted by ChEA3 tool. **(B)** Bar-graph showing number of targets out of oncogenic metabolic hubs in bladder cancer that are predicted to be regulated by each transcription factor in ChEA3 tool. **(C)** Bar-graph showing number of USF2 binding sites in genes from Metab-GS predicted by ChIPBase3 tool. **(D)** Bar-graph showing USF2 binding score for gene from Metab-GS calculated by Cistrome_DB tool. **(E)** Dot-plot showing changes in USF2 expression Z-score among low, medium and highly aggressive tumor groups in bladder cancer patients from TCGA database. **(F)** GSEA showing the enrichment of “Osman_Bladder_Cancer_Up” gene set between low and high USF2 expressing tumors in bladder cancer patients from TCGA database. **(G)** Kaplan-Meier survival plot showing survival analysis based on low and high USF2 expression in bladder cancer patients from TCGA database. **(H,I)** GSEA showing the enrichment of “GOCC_NuRD_Complex” **(H)** and “Senese_HDAC1_and_HDAC2_Targets_Up” **(I)** gene sets between low and high tumor aggressiveness groups in bladder cancer patients from TCGA database. **(J)** GSEA showing the enrichment of “Lindgren_Bladder_Cancer_Cluster_2B” gene set between low and high USF2-NuRD-Complex expression in bladder cancer patients from TCGA database. **(K,L)** Dot-plots showing correlation of Metab-OG-GS score with USF2-NuRD-Complex score **(K)** and HDAC1/2 targets score **(L)** in bladder cancer patients from TCGA database. NES, normalized enrichment score; Up, upregulated. **p < 0.01, ns: non-significant.

Next, we hypothesized that oncogenic metabolic hubs in Metab-GS may be regulated via transcription factor. To this end, we looked for TFs in ChEA3 tool, and found USF2 as top predicted transcription factor regulating oncogenic metabolic hubs in bladder cancer (Figures 5A,B). Data from ChIPBase also predicted multiple USF2 binding sites in oncogenic metabolic hubs but not in tumor suppressor metabolic hubs (Figure 5C). Data from Cistrome_DB also showed higher USF2 binding scores to oncogenic metabolic hubs, but these scores were minimal to none for tumor suppressor metabolic hubs in bladder cancer (Figure 5D) suggesting USF2 as key transcription factor to regulate oncogenic metabolic hubs in bladder cancer. Interestingly, USF2 expression was downregulated in aggressive disease state (Figure 5E) and genes upregulated in bladder cancer were enriched in patients having low USF2 expression (Figure 5F). In addition, high USF2 expression was associated with better survival in bladder cancer patients (Figure 5G).

Based on these interesting finding, we hypothesized that whatif USF2 downregulates oncogenic metabolic hubs in bladder cancer. To this end, we found that USF2 can bind to and get co-recruited with nucleosome remodeling and deacetylase (NuRD) complex at genomic regions and repress gene expression (Kim et al., 2024). NuRD complex is a multi-protein is a multi-protein chromatin remodeling complex that couples ATP-dependent chromatin remodeling with histone deacetylation and play key role in gene expression particularly through histone deacetylase (HDAC1 and HDAC2)-driven transcriptional repression (Lai and Wade, 2011). Notably, NuRD complex was also enriched (more active) in less aggressive bladder cancer tumors compared to highly aggressive ones (Figure 5H). Aligning with this, targets of HDA1/2 were enriched in highly aggressive bladder cancer tumors compared to less aggressive ones (Figure 5I). Moreover, bladder cancer-related genes were also enriched in tumors having low USF2-NurRD complex scores compared to those having high USF2-NuRD complex scores (Figure 5J). Lastly, oncogenic metabolic hubs in bladder cancer were negatively correlated with USF2-NuRD complex scores (Figure 5K) whereas positively correlated with HDAC1/2 target scores (Figure 5L). Overall, these findings suggest that USF2 regulated the expression of oncogenic metabolic hubs in bladder cancer via NuRD-complex driven histone deacetylation, and its downregulation leads to aggressive disease state and poor survival in bladder cancer patients.

### 3.5 USF2-NuRD complex/Metab-GS axis determines inflammatory tumor environment along with Warburg effect in bladder cancer

**FIGURE 6:**
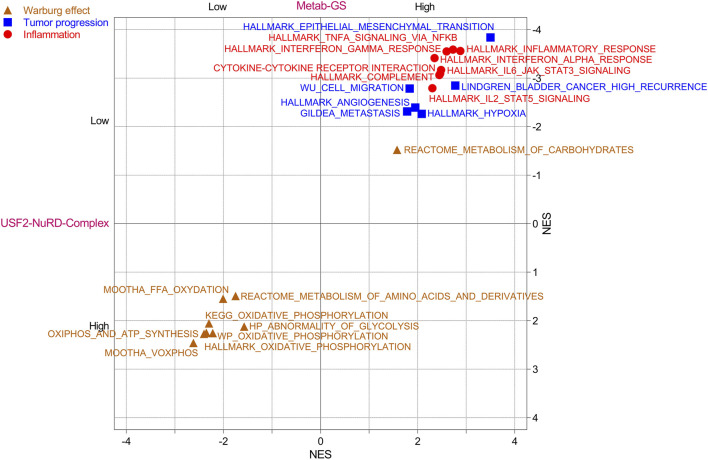
USF2-NuRD complex/Metab-GS axis determines inflammatory tumor environment along with Warburg effect in bladder cancer. Dot-plot showing the overlapping results of GSEA (normalized enrichment score) conducted between low vs. high Metab-GS score and between low vs. high USF2-NuRD-Complex score expressing tumors in bladder cancer patients from TCGA database, using gene sets related to Warburg effect (Brown), tumor progression (Blue) and inflammation (Red).

Cancer cell tend to rely more on glycolysis, and limit energy production through oxidative phosphorylation, a phenomenon known as Warburg effect, a key event of rapid proliferation and tumor progression. To this end, we tested whether USF2-NuRD complex/Metab-GS axis is associated with metabolic reprogramming in bladder cancer, and found that different oxidative phosphorylation-related gene signatures were enriched in patients having high USF2-NuRD complex scores and low Metab-GS scores (Figure 6, Brown) suggesting that Metab-GS is associated with Warburg effect in bladder cancer. On the other hand, different tumor progression-related gene signatures were enriched in patients having low USF2-NuRD complex scores and high Metab-GS scores (Figure 6, Blue) suggesting that Metab-GS is associated with tumor aggressiveness and progression in bladder cancer.

Inflammation ensues as tumor become aggressive. We next investigated whether the USF2-NuRD complex/Metab-GS axis correlates with the inflammatory landscape of aggressive bladder tumors. Our analysis revealed that several inflammation-associated gene signatures are significantly enriched in patients exhibiting low USF2-NuRD complex scores alongside high Metab-GS scores (Figure 6, Red), suggesting that Metab-GS is associated with inflammatory tumor environment in bladder cancer. In order to further validate this notion, we checked the expression of 188 inflammatory mediators representing various groups, including chemokine (C-C motif) ligands/chemokine (C-C motif) receptors (CCLs.CCRs), chemokine (C-X-C motif) ligands/chemokine (C-X-C motif) receptors (CXCLs/CXCRs), interferons/interferon regulatory factors (IFNs/IRFs), interleukins/interleukin receptors (ILs/ILRs), tumor necrosis factors/tumor necrosis factor receptors (TNFs/TNFRs), in patients with low and high Metab-GS scores. As a result, we found that 121 of these inflammatory mediators are differentially expressed between the two patient groups with 107 being highly expressed in patients having high METAB-GS scores (Supplementary Figures S4A–E). These findings further confirm that Metab-GS is associated with inflammatory tumor environment in bladder cancer.

### 3.6 Metab-GS determines aggressive disease state in bladder cancer patients

**FIGURE 7:**
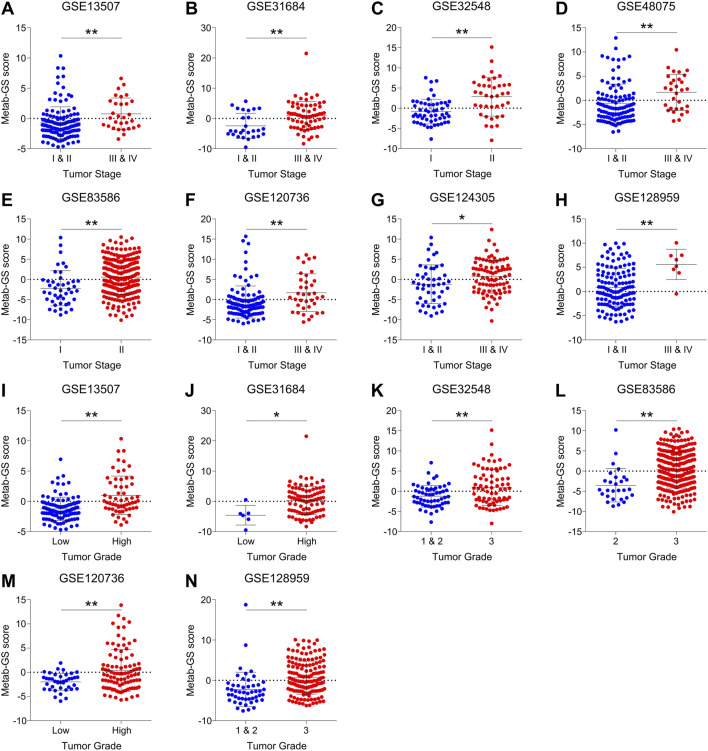
Metab-GS determines aggressive disease state in bladder cancer patients. **(A–H)** Dot-plot showing changes in Metab-GS scores between early and advanced tumor stage in bladder cancer patients from GSE13507 **(A)**, GSE31684 **(B)**, GSE32548 **(C)**, GSE48075 **(D)**, GSE83586 **(E)**, GSE120736 **(F)**, GSE124305 **(G)** and GSE128959 **(H)**. **(I–N)** Dot-plot showing changes in Metab-GS scores between early and advanced tumor grade in bladder cancer patients from GSE13507 **(I)**, GSE31684 **(J)**, GSE32548 **(K)**, GSE83586 **(L)**, GSE120736 **(M)** and GSE128959 **(N)**. **p < 0.01, *p < 0.05.

In order to validate our findings, we aimed to check whether Metab-GS is associated with disease progression and tumor aggressiveness in bladder cancer patients from independent GEO patient datasets. In this context, we found that high Metab-GS scores were associated with advanced tumor stage in bladder cancer patients from GSE13507 (Figure 7A), GSE31684 (Figure 7B), GSE32548 (Figure 7C), GSE48075 (Figure 7D), GSE83586 (Figure 7E), GSE120736 (Figure 7F), GSE124305 (Figure 7G) and GSE128959 (Figure 7H). Similarly, high Metab-GS scores were also associated with high tumor grades in bladder cancer patients from GSE13507 (Figure 7I), GSE31684 (Figure 7J), GSE32548 (Figure 7K), GSE83586 (Figure 7L), GSE120736 (Figure 7M) and GSE128959 (Figure 7N). These findings suggest that Metab-GS is associated with aggressive disease state in bladder cancer.

### 3.7 Metab-GS is associated with tumor progression, metastatic spread and disease recurrence in bladder cancer patients

**FIGURE 8:**
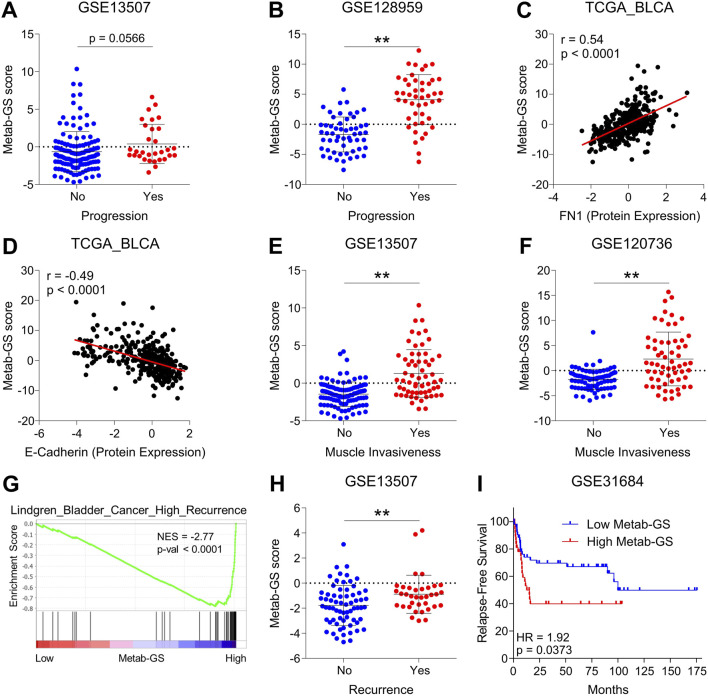
Metab-GS is associated with tumor progression, metastatic spread and disease recurrence in bladder cancer patients. **(A,B)** Dot-plot showing changes in Metab-GS scores between progressing and non-progressing tumors in bladder cancer patients from GSE13507 **(A)**, GSE128959 **(B)**. **(C,D)** Dot-plot showing correlation of Metab-GS score with protein expression of FN1 **(C)** and E-Cadherin **(D)** in bladder cancer patients from TCGA database. **(E,F)** Dot-plot showing changes in Metab-GS scores between muscle invasive and non-muscle invasive tumors in bladder cancer patients from GSE13507 **(E)** and GSE120736 **(F)**. **(G)** GSEA showing enrichment of “Lindgren_Bladder_Cancer_High_Recurrence” gene set between low and high Metab-GS score expression tumors in bladder cancer patients from TCGA database. **(H)** Dot-plot showing changes in Metab-GS scores in primary tumors from bladder patients from GSE13507 who experienced or did not experience disease recurrence. **(I)** Kaplan-Meier survival plot showing relapse-free survival analysis based on low and high Metab-GS score in bladder cancer patients from GSE31684. NES, normalized enrichment score. **p < 0.01.

Next, we tested whether Metab-GS is associated with tumor progression and metastatic spread in bladder cancer patients. In this context, we found that high Metab-GS scores are associated with tumor progression in bladder cancer patients from GSE13507 (Figure 8A) and GSE128959 (Figure 8B). In addition, Metab-GS scores are positively correlated with protein expression of mesenchymal marker, FN1 (Figure 8C), and positively correlated with epithelial marker, E-cadherin (Figure 8D) suggesting that Metab-GS correlates with EMT-driven tumor progression. Furthermore, High Metab-GS scores are also associated with muscle invasiveness in bladder cancer patients from GSE13507 (Figure 8E) and GSE120736 (Figure 8F). Moreover, gene set associated with high bladder cancer recurrence is also enriched in patients having high Metab-GS scores compared to those having low Metab-GS scores (Figure 8G). Similarly, high Metab-GS scores were associated with disease recurrence in bladder cancer patients from GSE13507 (Figure 8H). Moreover, high Metab-GS scores are associated with poor relapse-free survival in bladder cancer patients from GSE31684 (Figure 8I). In total, Metab-GS is associated with tumor progression, metastatic spread and disease recurrence in bladder cancer patients.

### 3.8 Metab-GS is associated with poor survival in bladder cancer patients

**FIGURE 9:**
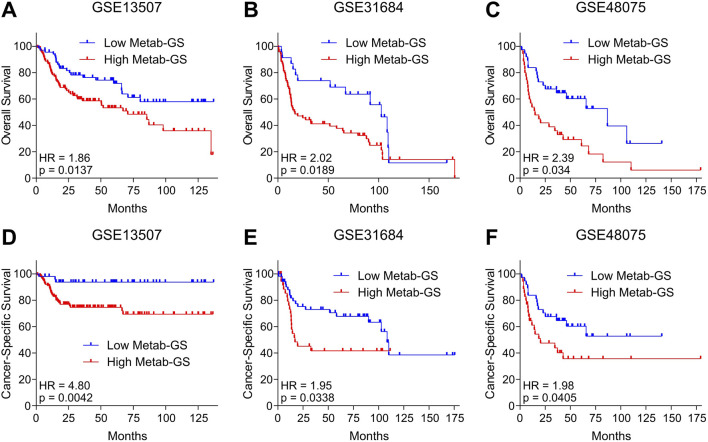
Metab-GS is associated with poor survival in bladder cancer patients. **(A–C)** Kaplan-Meier survival plot showing overall survival analysis based on low and high Metab-GS score in bladder cancer patients from GSE13507 **(A)**, GSE31684 **(B)** and GSE48075 **(C)**. **(D–F)** Kaplan-Meier survival plot showing cancer-specific survival analysis based on low and high Metab-GS score in bladder cancer patients from GSE13507 **(D)**, GSE31684 **(E)** and GSE48075 **(F)**.

In order to validate our findings that Metab-GS is associated with poor survival in bladder cancer patients, we tested survival association in independent GEO datasets. In this line, we found that high Metab-GS scores are associated with poor overall survival in bladder cancer patients from GSE13507 (Figure 9A), GSE31684 (Figure 9B) and GSE48075 (Figure 9C). Notably, high Metab-GS scores are also associated with poor cancer-specific survival in bladder cancer patients from GSE13507 (Figure 9D), GSE31684 (Figure 9E) and GSE48075 (Figure 9F) as well, confirming that Metab-GS is associated with poor survival in bladder cancer patients.

## 4 Discussion

By leveraging bladder cancer transcriptomic data from TCGA database, here, we first systematically screened for metabolic genes whose expression patterns correlated with tumor aggressiveness, tumor stage, and patient survival. Later, through advanced network construction and random walk analyses, we distilled these targets into a concise seven-gene metabolic signature (Metab-GS) comprising five oncogenic and two tumor suppressor metabolic hubs. Subsequent pathway enrichment revealed that these genes are centrally involved in key processes such as glycolytic reprogramming, extracellular matrix remodeling, and the promotion of an inflammatory tumor microenvironment. Notably, we observed that epigenetic modifications, specifically, DNA hypermethylation of tumor suppressor genes, and impaired USF2-NuRD complex-driven deacetylation and subsequent increased expression of oncogenic metabolic hubs collectively contribute to disease progression. Metab-GS not only drives glycolytic reprogramming, extracellular matrix remodeling, and the promotion of an inflammatory tumor microenvironment enriched with inflammatory mediators, but is also associated with tumor aggressiveness and progression, advanced disease stage, metastatic spread, disease recurrence, and poor overall and cancer-specific survival in bladder cancer patients as validated by independent GEO datasets.

The advent of high-throughput omics technologies has revolutionized cancer research, offering unprecedented insight into the molecular intricacies that drive tumor development and progression (Akhoundova and Rubin, 2022). In addition, aligning clinical features and patient survival data with gene expression profiles enables a reverse-engineering strategy, wherein observable tumor characteristics are traced back to their underlying transcriptomic patterns. This method provides crucial insights into how molecular alterations drive the initiation, progression, and aggressiveness of tumors (Li et al., 2021; Sanguedolce and Zanelli, 2022). In this study, metabolism-focused strategy that integrates tumor aggressiveness-related differential expression with survival analyses to dissect the metabolic processes underlying bladder cancer using transcriptomic data from The Cancer Genome Atlas (TCGA) and validated our findings using multiple independent GEO cohorts to dissect the metabolic reprogramming that fuels bladder cancer progression. Our multi-dimensional approach led to the identification of a distinct metabolic hubs that not only correlates with tumor aggressiveness (Figures 1–3) but also serves as a robust prognostic indicator of advanced disease and poor survival in bladder cancer patients (Figures 7–9).

A central finding of our work is the characterization of the Metab-GS, a seven-gene signature composed of both oncogenic and tumor suppressor metabolic hubs. The oncogenic components, namely, ALDH1B1, ALDH1L2, CHSY1, CSGALNACT2, and GPX8, were progressively upregulated in more aggressive tumors, while the tumor suppressor hubs FBP1 and HPGD were downregulated as tumor aggressiveness increased (Figure 3A). As discussed earlier, the perturbations in genes in Metab-GS have been associated with diverse cancer-promoting mechanisms in different cancer types effect (Zeng et al., 2018; Sun et al., 2021; Wu et al., 2021; Li et al., 2022; Xu et al., 2022; Lee and Vousden, 2024; Tsochantaridis et al., 2024). Elevated expression of ALDH1B1 and ALDH1L2 is particularly notable, as these enzymes are implicated in enhancing glycolytic flux and supporting one-carbon metabolism, thereby promoting the biosynthesis of nucleotides and sustaining rapid cell proliferation (Lee and Vousden, 2024; Tsochantaridis et al., 2024). Conversely, downregulation of FBP1 and HPGD, coupled with their hypermethylation, reinforces the metabolic shift toward glycolysis and potentiates prostaglandin-driven inflammation, both of which are key drivers of tumor progression (Sun et al., 2021; Xu et al., 2022).

DNA hypermethylation of tumor suppressor genes leads to their transcriptional silencing, effectively removing critical checks on the signaling pathways that drive tumor growth in different cancer types, including bladder cancer (Li et al., 2025). Our findings demonstrate a strong inverse correlation between DNA methylation levels at FBP1 and HPGD loci and their gene expression, thereby linking epigenetic dysregulation directly to the tumor aggressiveness in bladder cancer (Figure 4). These observations are consistent with prior studies showing that epigenetic modifications can serve as pivotal modulators of cancer metabolism in bladder cancer (Loras and Segovia, 2021). The selective hypermethylation of these key metabolic regulators may thus represent both a biomarker for aggressive disease and a potential therapeutic target. In parallel with DNA hypermethylation-based epigenetic regulation-driven downregulation of tumor suppressor metabolic hubs, our study also highlights the role of loss of deacetylation-driven transcriptional control of oncogenic metabolic hubs in driving metabolic reprogramming in bladder cancer (Figure 5). Through comprehensive transcription factor prediction analyses and binding site evaluations, we identified USF2 as a key regulator of the oncogenic metabolic hubs. USF2 has been previously implicated in various cellular processes, and particularly, has been shown to interact with HDAC1, recruiting NuRD complex and regulating the expression of lysosomal genes (Kim et al., 2024). This mechanistic insight is particularly compelling, as it suggests that modulating the activity of USF2 or the NuRD complex may restore the balance between oncogenic and tumor suppressor metabolic pathways, thereby impeding tumor growth.

Inflammation is another critical facet of bladder cancer biology that interplays with metabolic reprogramming. Chronic inflammation is known to drive tumorigenesis and promote an immunosuppressive microenvironment that supports cancer progression (Gakis, 2014; Greten and Grivennikov, 2019). In our study, we observed that high Metab-GS scores correlate with increased expression of inflammatory gene signatures (Figure 6). This inflammatory milieu not only sustains the metabolic alterations but also facilitates processes such as EMT, which is associated with enhanced invasiveness and metastatic potential (Figure 6) (Huang et al., 2022). The link between metabolic reprogramming and inflammation is further underscored by the finding that downregulation of HPGD, a key enzyme involved in prostaglandin degradation, leads to heightened prostaglandin signaling and inflammatory responses (Sun et al., 2021). These data highlight a vicious cycle in which metabolic dysregulation exacerbates inflammatory signaling, which in turn reinforces the metabolic state favorable to tumor growth and spread.

Our work is also notable for its translational relevance. The Metab-GS not only delineates the molecular landscape of bladder cancer but also serves as a potent prognostic tool. High Metab-GS scores were consistently associated with advanced tumor stages, higher grades, increased muscle invasiveness, and poor overall as well as cancer-specific survival in multiple independent patient cohorts (Figures 7–9). This is in line with the findings that metabolic genes can serve as biomarkers of disease in bladder cancer (Petrella et al., 2021), suggesting that the Metab-GS could be developed into a clinically useful biomarker to stratify patients according to their risk profile and potentially guide therapeutic decisions.

Despite the comprehensive integrative analysis performed in this study, several limitations should be acknowledged. Firstly, our findings are primarily based on bioinformatic analyses of publicly available datasets; thus, experimental validation *in vitro* and *in vivo* is needed to confirm the mechanistic roles of the identified metabolic hubs and their epigenetic regulation. Secondly, while we established correlations between DNA methylation, USF2-NuRD complex activity, and metabolic gene expression, direct causal relationships remain to be demonstrated. Thirdly, although multiple independent cohorts validated the prognostic value of Metab-GS, prospective clinical studies are required to evaluate its utility in patient stratification and therapy guidance. Addressing these limitations in future research will be essential to translate our findings into effective therapeutic strategies for bladder cancer. Looking ahead, our study opens several promising avenues for future research. First, the reversal of epigenetic silencing, potentially through the use of DNA methyltransferase inhibitors (Nunes and Henrique, 2020), could restore the expression of tumor suppressor metabolic hubs such as FBP1 and HPGD, thereby rebalancing cellular metabolism and limiting tumor progression. Second, therapeutic strategies aimed at modulating the USF2-NuRD axis could reinstate the repression of oncogenic metabolic targets (Kim et al., 2024). Third, given the intimate connection between metabolic reprogramming and inflammation, combination therapies that target both pathways might provide synergistic benefits, especially in aggressive bladder cancers (Scholtes et al., 2021).

## 5 Conclusion

Our study provides a comprehensive framework linking epigenetic dysregulation with metabolic reprogramming in bladder cancer. The identification of the Metab-GS along with the regulation of tumor suppressor metabolic hubs via DNA hypermethylation and the regulation of oncogenic metabolic hubs by the USF2-NuRD complex offers new insights into the mechanisms driving tumor aggressiveness and poor clinical outcomes in bladder cancer. These findings not only enhance our understanding of bladder cancer biology but also pave the way for the development of innovative diagnostic and therapeutic strategies. By targeting the metabolic vulnerabilities of bladder cancer cell, through modulation of epigenetic regulators, it may be possible to curb tumor progression and improve patient survival, ultimately reducing the burden of this challenging disease.

## Data Availability

The datasets presented in this study can be found in online repositories. The names of the repository/repositories and accession number(s) can be found in the article/Supplementary Material.
