# Supplementary material for: Epigenetic dysregulation-induced metabolic reprogramming fuels tumor progression in bladder cancer
Source: Front Mol Biosci. 2025 Jun 23;12:1602700. doi: 10.3389/fmolb.2025.1602700 (PMC12229847; doi:10.3389/fmolb.2025.1602700)
Supplement: Supplementary file 1 [file DataSheet1.pdf]

Epigenetic dysregulation-induced metabolic reprogramming fuels tumor progression in bladder cancer

Supplementary figures and their legends

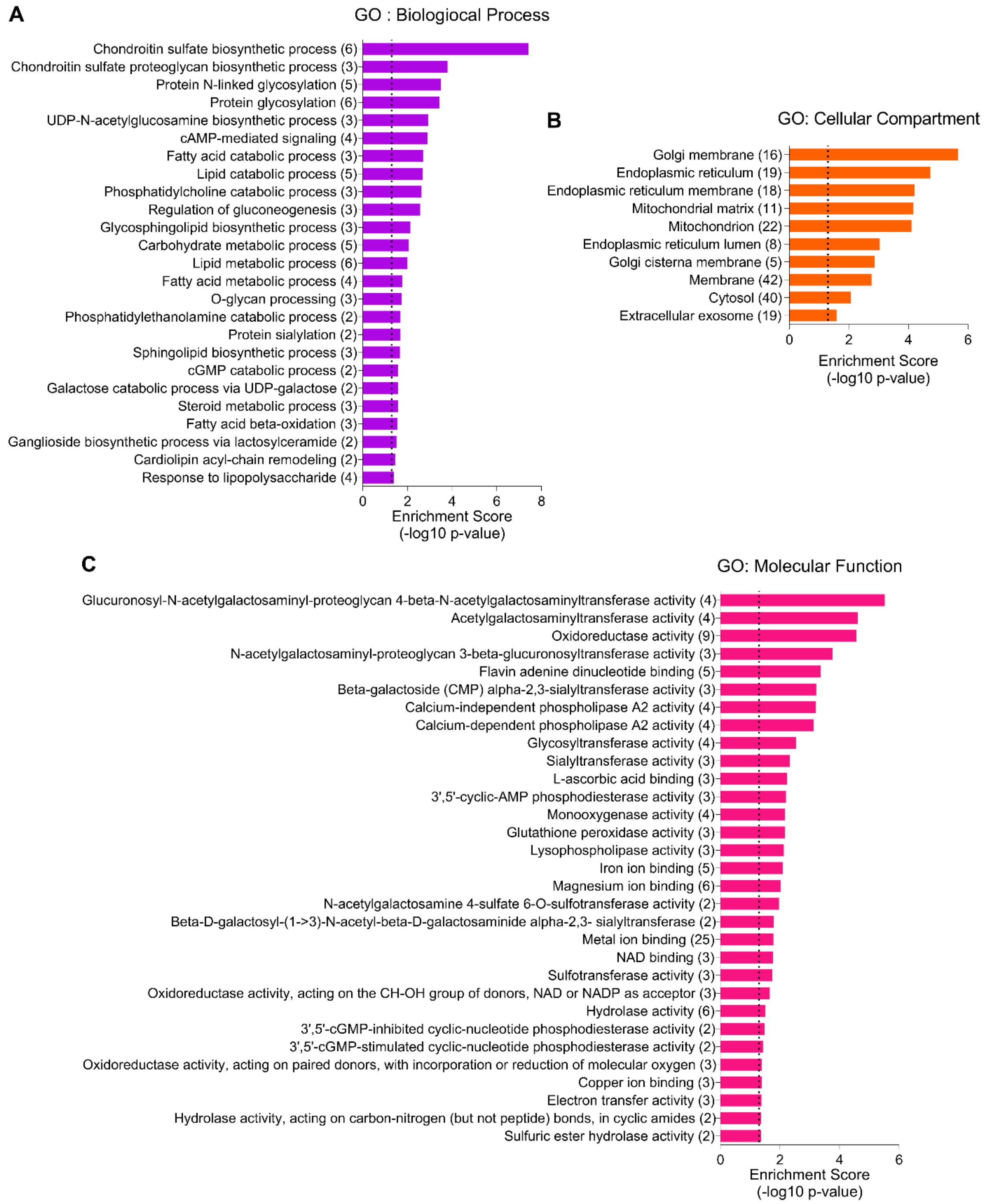

Figure S1: GO functional enrichment analysis. (A-C) Bar-graphs showing results of GO pathway enrichment analysis (Biological process (A), Cellular compartment (B) and Molecular function (C)) using common metabolic targets identified in Figure 1A and B.

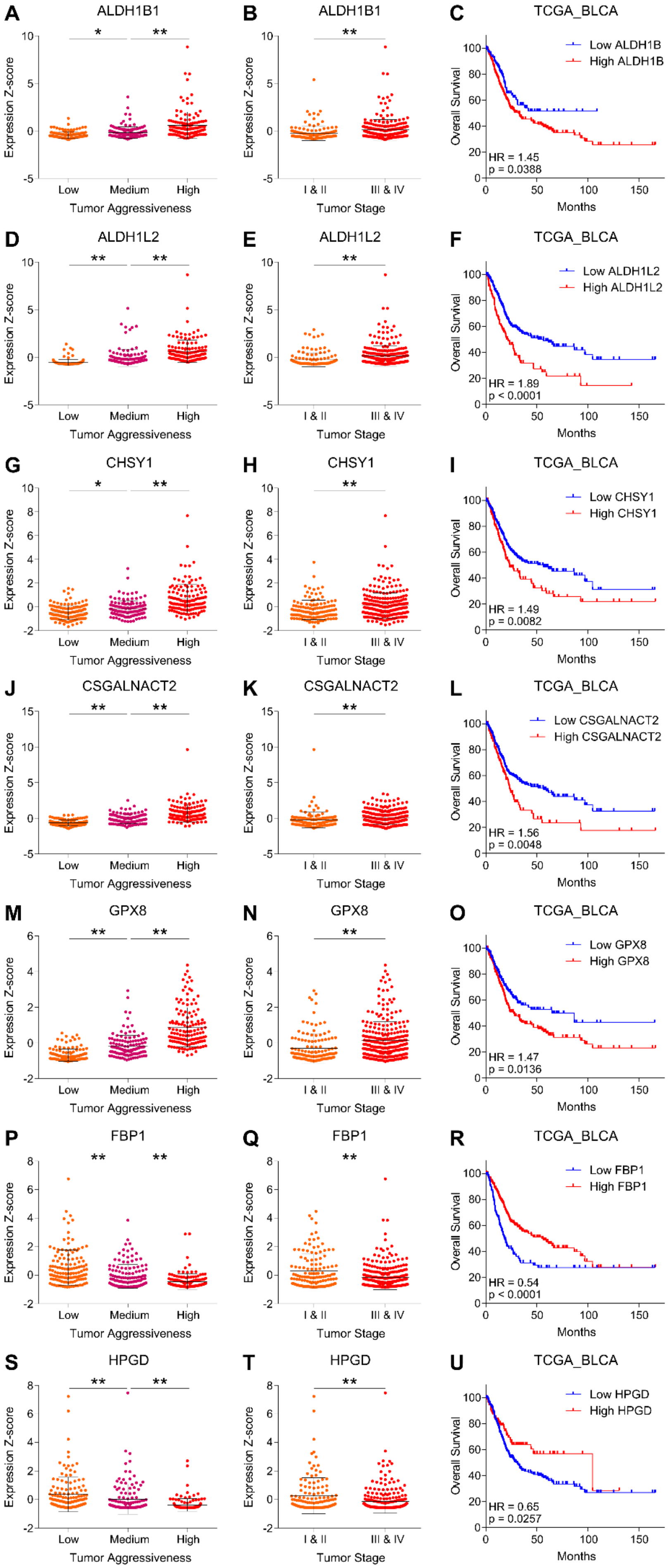

A
ALDH1B1
Expression Z-score
Low
Medium
High
Tumor Aggressiveness
B
ALDH1B1
Expression Z-score
I & II
III & IV
Tumor Stage
C
TCGA_BLCA
Low ALDH1B
High ALDH1B
Overall Survival
HR = 1.45
p = 0.0388
Months
D
ALDH1L2
Expression Z-score
Low
Medium
High
Tumor Aggressiveness
E
ALDH1L2
Expression Z-score
I & II
III & IV
Tumor Stage
F
TCGA_BLCA
Low ALDH1L2
High ALDH1L2
Overall Survival
HR = 1.89
p < 0.0001
Months
G
CHSY1
Expression Z-score
Low
Medium
High
Tumor Aggressiveness
H
CHSY1
Expression Z-score
I & II
III & IV
Tumor Stage
I
TCGA_BLCA
Low CHSY1
High CHSY1
Overall Survival
HR = 1.49
p = 0.0082
Months
J
CSGALNACT2
Expression Z-score
Low
Medium
High
Tumor Aggressiveness
K
CSGALNACT2
Expression Z-score
I & II
III & IV
Tumor Stage
L
TCGA_BLCA
Low CSGALNACT2
High CSGALNACT2
Overall Survival
HR = 1.56
p = 0.0048
Months
M
GPX8
Expression Z-score
Low
Medium
High
Tumor Aggressiveness
N
GPX8
Expression Z-score
I & II
III & IV
Tumor Stage
O
TCGA_BLCA
Low GPX8
High GPX8
Overall Survival
HR = 1.47
p = 0.0136
Months
P
FBP1
Expression Z-score
Low
Medium
High
Tumor Aggressiveness
Q
FBP1
Expression Z-score
I & II
III & IV
Tumor Stage
R
TCGA_BLCA
Low FBP1
High FBP1
Overall Survival
HR = 0.54
p < 0.0001
Months
S
HPGD
Expression Z-score
Low
Medium
High
Tumor Aggressiveness
T
HPGD
Expression Z-score
I & II
III & IV
Tumor Stage
U
TCGA_BLCA
Low HPGD
High HPGD
Overall Survival
HR = 0.65
p = 0.0257
Months

Figure S2: Metabolic hubs determines aggressive disease state and poor survival in bladder cancer. (A-G) Dot-plots showing changes in expression of seven metabolic hubs among low, medium and highly aggressive tumor groups in bladder cancer patients from TCGA database. (H-N) Dot-plots showing changes in expression of seven metabolic hubs between early and advanced stage tumors in bladder cancer patients from TCGA database. (O-U) Kaplan-Meier survival plots showing overall survival analysis based on low and high expression of seven metabolic hubs in bladder cancer patients from TCGA database. **: $p<0.01$, *: $p<0.05$.

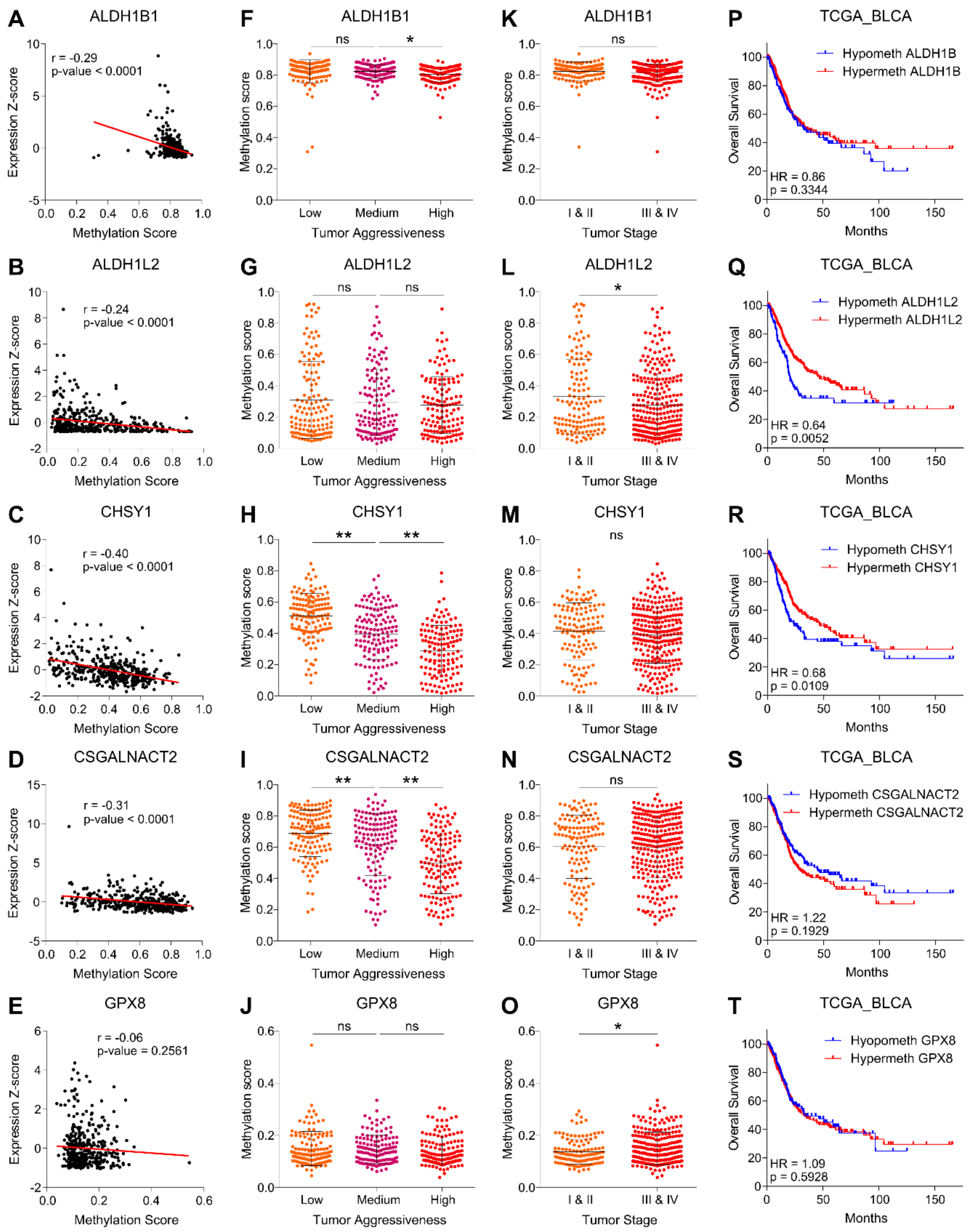

Figure S3: DNA hypermethylation of oncogenic metabolic hubs is not associated with disease progression in bladder cancer. (A-E) Dot-plots showing correlation between expression Z-score and DNA methylation score of oncogenic metabolic hubs in bladder cancer patients from TCGA database. (F-J) Dot-plots showing changes in methylation score of oncogenic metabolic hubs among low, medium and highly aggressive tumor groups in bladder cancer patients from TCGA database. (K-O) Dot-plots showing changes in methylation score of oncogenic metabolic hubs between early and advanced stage tumors in bladder cancer patients from TCGA database. (P-T) Kaplan-Meier survival plot showing survival analysis based on low and high methylation score of oncogenic metabolic hubs in bladder cancer patients from TCGA database. **: p<0.01, *: p<0.05, ns: non-significant.

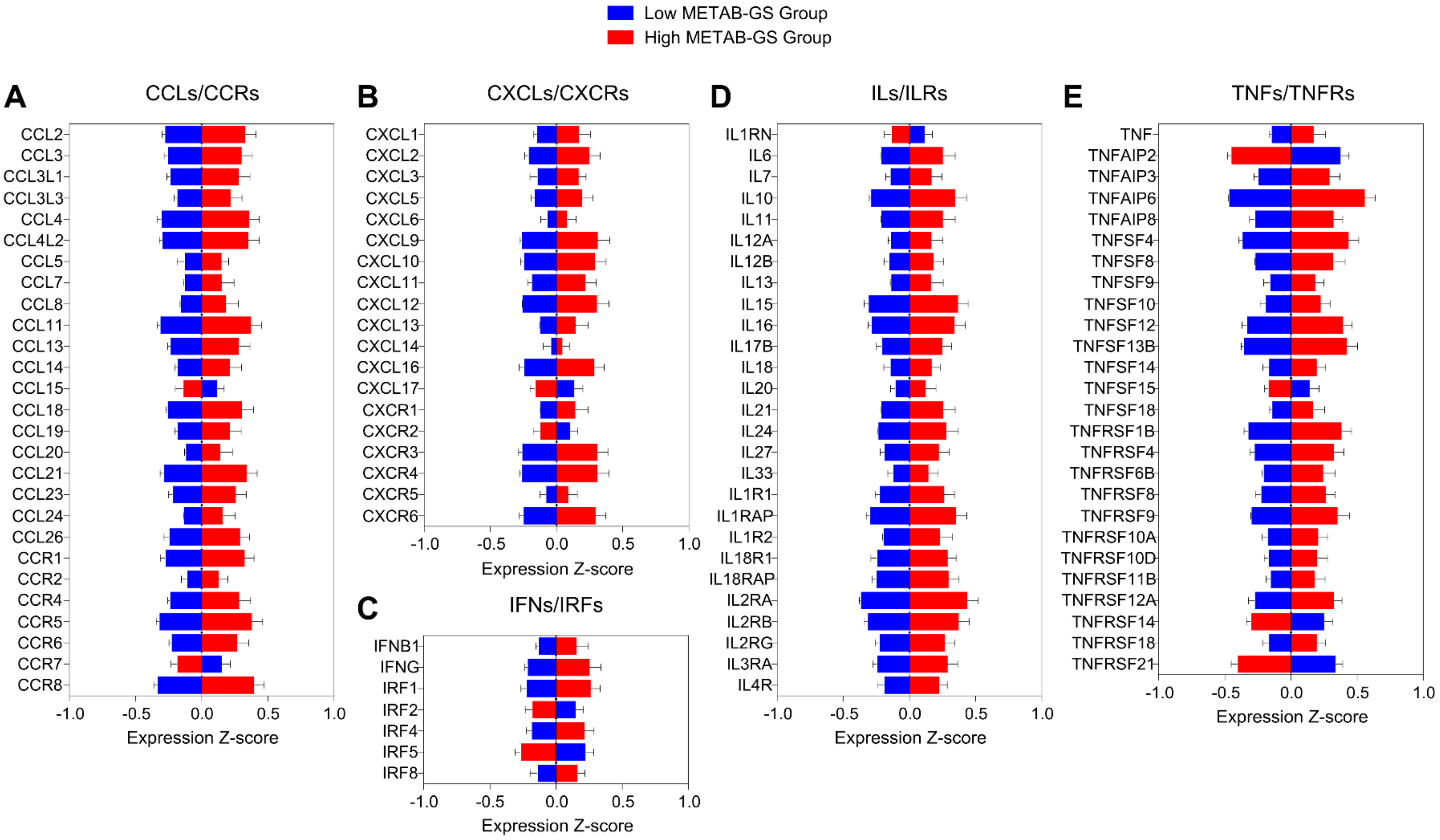

Figure S4: Inflammatory mediators are enriched in patients having high METAB-GS scores. (A-E) Bar-graphs showing differential expression analysis of various groups of inflammatory mediators including CCLs/CCRs (A), CXCLs/CXCRs (B), IFNs/IRFs (C), ILs/ILRs (D) and TNFs/TNFRs in BLCA patients having low or high METAB-GS scores in TCGA database. Out of total 188 inflammatory mediators belonging to these five groups, only 121 with significant differential expression are shown in these graphs.
